# A Review of the Bromodomain and Extraterminal Domain Epigenetic Reader Proteins: Function on Virus Infection and Cancer

**DOI:** 10.3390/v16071096

**Published:** 2024-07-08

**Authors:** Mengli Wu, Guiquan Guan, Hong Yin, Qingli Niu

**Affiliations:** 1 State Key Laboratory for Animal Disease Control and Prevention, College of Veterinary Medicine, Lanzhou University, Lanzhou Veterinary Research Institute, Chinese Academy of Agricultural Sciences, Lanzhou 730000, China; 15238649312@163.com (M.W.); guanguiquan@caas.cn (G.G.); yinhong@caas.cn (H.Y.); 2 African Swine Fever Regional Laboratory of China (Lanzhou), Gansu Province Research Center for Basic Disciplines of Pathogen Biology, Lanzhou 730046, China; 3 Jiangsu Co-Innovation Center for the Prevention and Control of Important Animal Infectious Disease and Zoonosis, Yangzhou University, Yangzhou 225009, China

**Keywords:** BET family, transcription, viral infection, inhibitors, cancer

## Abstract

The BET (bromodomain and extraterminal domain) family of proteins, particularly BRD4 (bromodomain-containing protein 4), plays a crucial role in transcription regulation and epigenetic mechanisms, impacting key cellular processes such as proliferation, differentiation, and the DNA damage response. BRD4, the most studied member of this family, binds to acetylated lysines on both histones and non-histone proteins, thereby regulating gene expression and influencing diverse cellular functions such as the cell cycle, tumorigenesis, and immune responses to viral infections. Given BRD4’s involvement in these fundamental processes, it is implicated in various diseases, including cancer and inflammation, making it a promising target for therapeutic development. This review comprehensively explores the roles of the BET family in gene transcription, DNA damage response, and viral infection, discussing the potential of targeted small-molecule compounds and highlighting BET proteins as promising candidates for anticancer therapy.

## 1. Introduction

Epigenetics explores the mechanisms of heritable changes in gene expression that occur without alterations to the DNA sequence [1,2]. These modifications, primarily driven by processes such as DNA methylation and histone modification, regulate gene activity crucial for development and adaptation to environmental changes [3]. Central to these regulatory mechanisms is the BET (bromodomain and extraterminal) family of proteins, with bromodomain-containing protein 4 (BRD4) playing a pivotal role. BRD4 modulates the chromatin structure and function, thus influencing gene expression and impacting a wide range of cellular processes [4].

BRD4 specifically interacts with acetylated lysines on histone tails, a crucial interaction that supports transcriptional activation, cell cycle progression, and cellular responses to DNA damage [5]. This interaction extends beyond basic cellular functions such as proliferation and differentiation, to playing substantial roles in the pathology of diseases like cancer and inflammatory disorders [6,7]. As the most extensively studied member of the BET family, BRD4’s ability to bind to both histones and non-histone proteins makes it a critical regulator of gene expression across diverse biological contexts, from cell cycle regulation to tumorigenesis, and its involvement in immune responses to viral infections [8,9].

This broad functionality underscores the potential of BRD4 to impact various pathological conditions, establishing it as an attractive target for therapeutic intervention. This review aims to elucidate the multifaceted roles of BRD4 within the BET family, exploring its contributions to transcription regulation, DNA damage repair, and viral pathogenesis. Additionally, the therapeutic potential of small-molecule compounds targeting BRD4 and highlights of the significance of BET proteins, particularly BRD4, in developing anticancer therapies are discussed. The discussion intends to reflect both the current state of research and explore future directions in this dynamic field.

## 2. Structure and Classification of the BET Family

The BET family of proteins, encompassing BRD2, BRD3, BRD4, and BRDT, are pivotal regulators of gene expression, mediating the crosstalk between chromatin structure and transcriptional control [10]. Characterized by their conserved structural domains, these proteins play critical roles in various cellular processes, including cell cycle progression, development, and the response to DNA damage [8,9].

**Figure 1:**
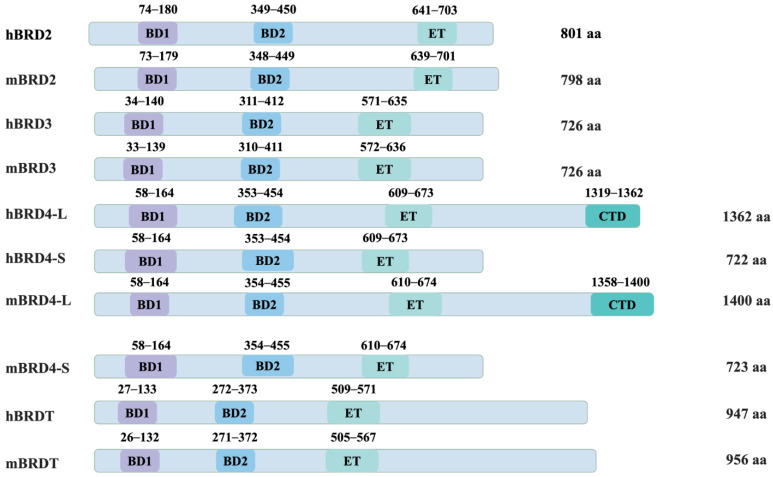
The structural domains of BET family proteins in humans and Mus musculus. Numbers represent the amino acid boundaries of each structural domain in each protein. Amino acid sequences were compared from publicly available information retrieved from the National Library of Medicine (NCBI) at hBRD2. UQL51212.1; mBRD2, NP_034368.2; hBRD3, NP_031397.1; mBRD3:NP_075825.3; hBRD4-L, NP_001366220.1; hBRD4-S, NP_001366221.1; mBRD4-L. AAL67833.1; mBRD4-S, AAL67834.1; hBRDT, AAB87862.1; and mBRDT, NP_473395.2. The BET family all contains two bromodomains, BD1 and BD2, and an additional terminal structural domain (ET). In addition to this, BRD4-L (long form) also contains a carboxy-terminal domain (CTD), which is not found in other BET family proteins.

A defining feature of BET proteins is the presence of two conserved bromodomains (BD1 and BD2) at their N-terminus, which are approximately 110 amino acids in length and function as epigenetic readers [11]. Bromodomains specifically recognize and bind acetylated lysine residues on histone tails, a post-translational modification associated with active chromatin and gene expression. This binding facilitates the recruitment of BET proteins to chromatin, where they exert their regulatory functions. In addition to the bromodomains, BET proteins possess an extra-terminal (ET) domain, which contributes to their functional diversity (Figure 1).

### 2.1. BRD2

BRD2, also known as RING3 or FSRG1, functions as part of a multiprotein complex of RNA Pol II transcriptional co-activators [12,13]. It plays a vital role in the cell cycle and chromatin remodeling and has been implicated in various developmental and physiological processes including neural tube closure, embryogenesis, adipogenesis, and immune response regulation [14,15,16]. BRD2 is essential for RNA polymerase II-mediated transcription elongation by modulating the chromatin structure and stabilizing the transcription machinery, ensuring efficient transcription and genomic stability. In the immune system, BRD2 is particularly important for B-cell-mediated class switch recombination, which is crucial for effective antibody production and adaptive immune responses [17]. It also regulates natural killer (NK) cell functions, influencing cytokine production and cytolytic activity. Furthermore, BRD2 is implicated in modulating the transcription of genes activated during viral infections, including those crucial for the interferon response integral to antiviral defense mechanisms. Significantly, BRD2 regulates the transcription of the angiotensin-converting enzyme 2 (ACE2), which is recognized as the primary receptor for the spike protein of SARS-CoV-2. This process facilitates the virus’s entry into human cells, highlighting BRD2’s pivotal role in the pathogenesis of SARS-CoV-2 infections [16].

### 2.2. BRD3

BRD3, identified also as ORFX or RING3L, shares critical functional characteristics with BRD2, notably in binding acetylated histones. Like BRD2, BRD3 promotes transcription by facilitating the interaction of RNA polymerase II with hyperacetylated nucleosomes [12,18]. Functionally, BRD3 plays a pivotal role in essential cellular processes such as cell cycle regulation and cellular differentiation and is implicated in the pathogenesis of various cancers [19,20]. Both BRD2 and BRD3 demonstrate histone chaperone activity by facilitating transcription independently of FACT (facilitated chromatin transcription) factors, highlighting their autonomous roles in chromatin dynamics [21]. Beyond interacting with histones, BRD3 directly engages with key transcription factors, notably influencing erythropoiesis by regulating the activity of GATA1 at erythroid gene promoters [22]. The extra-terminal (ET) domain of BRD3, a feature conserved within the BET family, facilitates its interaction with a broad array of protein–protein networks, including those involving viral proteins [23].

### 2.3. BRDT

BRDT, also known as BRD6 or CT9, plays a crucial role in gene expression regulation during the meiotic and post-meiotic phases [24]. It binds to acetylated lysine residues, a critical mechanism for regulating chromatin structure and epigenetic processes during meiosis [25]. BRDT also influences enhancer activity by modulating promoter-proximal pausing and the synthesis of enhancer RNAs (eRNAs). eRNAs are non-coding RNAs transcribed from enhancer regions of DNA, which play significant roles in the activation of gene expression by promoting chromatin accessibility and facilitating the assembly of transcription factors on target promoters. This function is crucially dependent on its interaction with the P-TEFb subunit CDK9, a key regulator in this pathway [26]. Predominantly expressed in the testis, BRDT is essential for male germ cell differentiation [27]. Mutations in BRDT are directly linked to male sterility, emphasizing its critical role in reproductive health. Additionally, BRDT oversees meiotic processes that are crucial for maintaining the genetic and epigenetic integrity of male gametes [25,28].

### 2.4. BRD4

BRD4, known alternatively as CAP, HUNK1, or MCAP, functions as a central figure in transcriptional and epigenetic regulation [29]. It is integral to the transcriptional machinery, facilitating RNA polymerase II-mediated transcriptional elongation by binding acetylated lysine residues on histones, which opens chromatin and enhances gene expression [30]. This role is particularly significant in the context of cancer, where BRD4 is frequently overexpressed and interacts with E2F transcription factors to regulate genes crucial for cell cycle progression and DNA replication [31,32]. The modulation of these processes by BRD4 highlights its potential as a therapeutic target, especially in MYC-driven and inflammatory cancers [33,34].

Beyond oncology, BRD4’s influence extends to viral pathogenesis, cardiovascular diseases, and immune regulation. It is involved in the life cycles of viruses like HIV HPV and SARS-CoV-2, affecting viral transcription processes and representing a potential target for antiviral therapies [35,36,37,38]. In cardiovascular health, BRD4 has been linked to conditions such as pulmonary arterial hypertension and heart failure, suggesting its roles in broader physiological and pathological processes [39,40].

BRD4’s functionality is further regulated by various post-translational modifications (PTMs), such as phosphorylation, ubiquitination, and acetylation, which impact its stability and interaction with other proteins and transcriptional coactivators [41,42]. These PTMs play critical roles in determining BRD4’s involvement in disease progression and the response to environmental stresses, pointing to its complex regulatory mechanisms in cellular function and stability.

## 3. BRD4 in Transcription

BRD4 exerts a profound influence on gene expression, impacting various stages of transcription—primarily during initiation and elongation. It plays a crucial role in relieving the promoter-proximal pausing of RNA polymerase II (Pol II), predominantly through recruiting the positive transcription elongation factor b (P-TEFb), which phosphorylates Pol II’s C-terminal domain (CTD) [29]. This phosphorylation is essential for transitioning Pol II from pausing to productive elongation [43,44,45].

Additionally, BRD4 enhances the transcriptional process through its interaction with acetylated histones at super-enhancer sites [46]. These interactions are critical for the regulation of genes that determine cell identity and are implicated in disease pathogenesis, particularly in cancer [47]. This binding facilitates a sustained active chromatin state, thereby promoting the transcription of downstream target genes.

Moreover, BRD4’s involvement extends to the integration of signaling pathways and transcriptional regulation [6,28,34]. It acts as a bridge between distal regulatory elements and core promoter regions by interacting with the mediator complex. This interaction facilitates the synchronized assembly of the preinitiation complex at promoter sites, enhancing the responsiveness of transcriptional initiation to developmental cues and environmental signals.

By modulating the chromatin structure through its histone acetylation activity and recruiting various transcriptional coactivators and elongation factors, BRD4 orchestrates a finely tuned regulatory network that governs transcriptional fidelity and dynamics [6,13]. This comprehensive regulatory role makes BRD4 a key therapeutic target, especially in oncology, where the modulation of transcription can influence cancer progression and treatment outcomes.

## 4. BRD4 in DNA Damage

DNA damage encompasses modifications to the DNA structure that impair its function, arising from various stressors such as oxidative or metabolic stress, and carcinogenic or genotoxic agents [48]. Such damage can lead to mutations, disrupt cellular processes, and activate DNA repair mechanisms or cell death pathways [49].

BRD4 is pivotal in regulating the DNA damage response (DDR), demonstrating dual functionality by both promoting and inhibiting DNA repair processes. This depends on the cellular context and specific challenges presented by DNA damage.

BRD4’s role in modulating replication-stress-induced DDR is marked by its interaction with acetylated chromatin, crucial during the interphase and mitosis, thereby helping to maintain genomic integrity. This interaction is essential not only for the normal progression of the cell cycle but also for the initiation of DNA repair processes during replication stress [50,51]. Additionally, BRD4 supports the resection and homologous recombination repair of DNA double-strand breaks, vital for accurate DNA repair mechanisms [52,53].

BRD4 can also attenuate DDR. Its inhibition or knockdown enhances the DNA damage checkpoint recovery speed and efficiency, suggesting a role in suppressing some DDR aspects. This suppression is linked to BRD4’s ability to promote a more closed chromatin structure, which might affect the phosphorylation of H2AX, an early DDR signaling marker [52].

Moreover, BRD4’s regulation extends to pre-replication factors such as CDC6, underscoring its importance in DNA replication checkpoint signaling. Inhibiting BRD4 reduces CHK1 phosphorylation and triggers aberrant DNA replication re-initiation, highlighting a complex role in genomic stability [54].

Arginine methylation further influences BRD4’s regulatory functions on transcription and DNA repair. This post-translational modification, mediated by PRMT2 and PRMT4, is pivotal for BRD4’s recruitment to acetylated chromatin and enhances its roles in transcription and DNA repair, particularly under DNA-damaging conditions [55].

BRD4’s intricate involvement in DDR, transcription regulation, and cancer biology not only underscores its vital cellular roles but also its potential as a therapeutic target in cancer treatment. Manipulating DDR pathways via BRD4 can decisively affect treatment outcomes. Continued research into BRD4’s diverse functions promises new therapeutic strategies targeting its activity in cancer and other diseases with genomic instability.

## 5. BRD4 in Virus Infection

**Figure 2:**
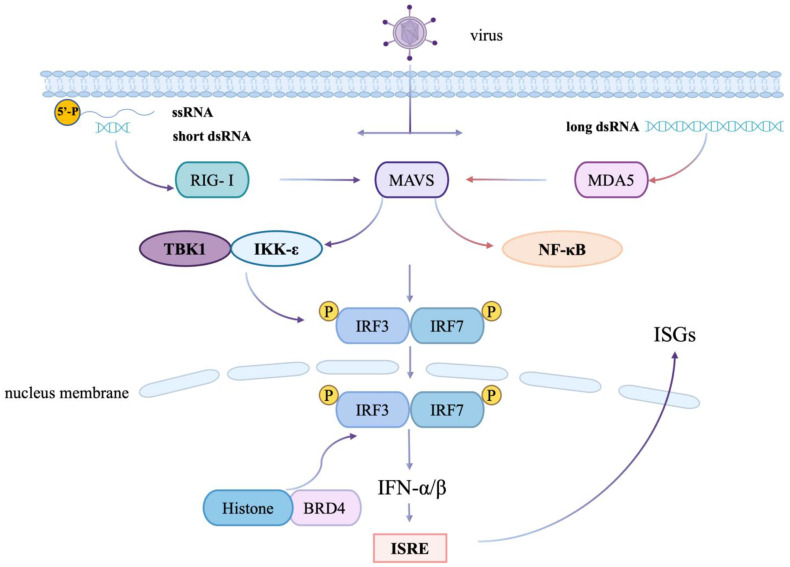
The role of RIG-I-like receptor signaling pathway in antiviral immunity. BRD4 mediates IRF and NF-κB-dependent innate response networks through RIG-I and TLR3 signaling pathways. BRD4 can also regulate viral infection by interacting with interferon-stimulated genes (ISGs) and viral proteins.

The immune response of the host is categorized into two main types: innate and adaptive immunity [56]. The innate immune response, serving as the initial defense against pathogens, is rapid and nonspecific [57]. This response involves various epigenetic mechanisms that control gene expression at multiple levels. Innate immunity detects pathogens through pattern recognition receptors (PRRs) present on both immune and non-immune cells, initiating broad immune signaling pathways [58] (Figure 2).

BRD4 plays a pivotal role in regulating the response to viral infections by influencing transcriptional pathways that affect both innate and adaptive immunity. Its primary function within innate immunity involves modulating the transcription of immune response genes via interactions with key transcription factors [59] (Table 1).

### 5.1. RNA Virus

#### 5.1.1. RSV

BRD4 plays a central role in the immune response to Respiratory Syncytial Virus (RSV) infections, particularly by orchestrating the regulation of gene expression in the innate immune system. During RSV infection, BRD4 modifies the transcriptional landscape, primarily influencing key signaling pathways such as the NF-kB and IRF families within the RIG-I-like receptor (RLR) pathway. This pathway is crucial for detecting RSV and orchestrating a robust immune defense, culminating in the production of type I and III interferons and pro-inflammatory cytokines, essential for combating the viral pathogens [60].

RD4’s role extends to enhancing the activity of promoters for critical antiviral defense genes such as IRF1, IRF7, and RIG-I, through its interactions with the acetylated lysines on histones, facilitating transcriptional elongation and enhancing gene expression critical to an effective antiviral response. In respiratory diseases like RSV, BRD4 functions as a potent co-activator of interferon-stimulated genes (ISGs), initiating pro-inflammatory responses essential for viral defense [61].

In respiratory diseases like Respiratory Syncytial Virus (RSV), BRD4 acts as a potent co-activator of ISGs during viral infections, initiating pro-inflammatory responses. The immune response begins with the activation of receptors like Toll-like receptor 3 (TLR-3) and RIG-I by viral-pathogen-associated molecular patterns (PAMPs), which then signal through transcription factors to trigger the innate immune response [62].

A key pathway involves the activation of a complex between RelA and BRD4 by PAMPs, which enhances BRD4’s enzymatic activity in respiratory cells, leading to acute inflammation. Among the transcription factors activated, NF-kB is essential for the expression of inflammatory chemokines and interferons. This process involves the release of RelA from cytoplasmic inhibitors, allowing it to specifically bind DNA at the BRD4 junction, enhanced further by nuclear-stress-induced phosphorylation [7,60].

The NF-kB-BRD4 complex plays a dual role by directly activating inflammatory chemokines and indirectly stimulating mucosal interferons through interaction with the IRF-RIG-I pathway, initiating lung adaptive immune responses. These intricate regulatory mechanisms underscore the potential for therapeutic strategies in managing respiratory inflammatory disorders.

#### 5.1.2. HIV

BRD4 is pivotal in the regulation of HIV transcription; thus, it stands as a key target for therapeutic strategies designed to manage HIV infection and control viral latency [63]. The primary mechanism by which BRD4 influences HIV transcription and latency is through its interaction with the positive transcription elongation factor b (P-TEFb) and the viral transactivator protein, Tat, which are essential for the elongation process of viral transcription. BRD4 competes with Tat for binding to P-TEFb, a critical interaction since P-TEFb facilitates the effective elongation of HIV transcription by RNA polymerase II. When BRD4 binds to P-TEFb, it impedes this elongation process, which is vital for viral replication [26].

Furthermore, BRD4’s interaction with chromatin, through its binding to acetylated lysines on histones H3 and H4 at the HIV long terminal repeat (LTR) site, plays a crucial role in modulating HIV latency. This interaction can lead to the repression or activation of HIV transcription, influenced by the dynamic state of histone modifications and the interplay with other transcriptional regulators [63].

BRD4’s role extends beyond simple interactions with P-TEFb and histones; it acts as a sophisticated epigenetic regulator within the HIV transcriptional context. Notably, BRD4 can both promote and inhibit HIV-1 transcription. It promotes latency by competitively inhibiting the Tat-induced activation of P-TEFb. Conversely, overexpression of the C-terminal region of BRD4 can block HIV-1 infection and interfere with the functional interaction between Tat and P-TEFb in various experimental models [64]. Moreover, the different isoforms of BRD4, such as BRD4L and BRD4S, display distinct functions: BRD4L enhances basal HIV-1 transcription in a Tat-independent manner by activating P-TEFb, while BRD4S acts as a transcriptional repressor that promotes viral latency by participating in the SWI/SNF chromatin remodeling complex [65]. These diverse roles underscore the complexity of BRD4’s involvement in HIV regulation and highlight its potential as a multifaceted target for therapeutic intervention.

#### 5.1.3. SARS-CoV-2

SARS-CoV-2, the causative agent of COVID-19, relies on crucial structural proteins—spike (S), nucleocapsid (N), membrane (M), and envelope (E)—for viral entry and replication [66]. Recent studies have demonstrated that BRD4, a member of the BET protein family, interacts with acetylated proteins, including the C-terminus of the SARS-CoV-2 E protein. This interaction is mediated through the bromodomains (BDs) of BRD4, which bind to the acetylated E protein, a modification attributed to the human acetyltransferase p300 [67]. Such interactions suggest a mechanism by which SARS-CoV-2 might hijack the host’s transcriptional machinery to promote viral replication and modulate innate immune responses.

Moreover, the regulation of ACE2 expression, the receptor for SARS-CoV-2, has been linked more specifically to BRD2. BRD2 inhibitors have been shown to decrease ACE2 expression and reduce SARS-CoV-2 infectivity in various cell models, suggesting that the manipulation of BET protein activity could impact both viral entry and the subsequent inflammatory response [38].

### 5.2. DNA Virus

#### 5.2.1. HPV

Among the proteins encoded by Human Papillomavirus (HPV), E2 is distinctive as a DNA-binding protein that orchestrates various facets of the virus’s life cycle [68]. BRD4 is identified as a critical host-interacting partner of E2, playing a significant role in the regulation of transcription, initiation of DNA replication, and viral genome maintenance [69]. The interaction between BRD4 and E2 is multifaceted, involving specific binding between E2 and several domains of BRD4—such as the C-terminal structural domain (CTM), phosphate-dependent interaction domain (PDID), and basic interaction domain (BID)—which are crucial for E2’s transcriptional regulatory activities [37,70].

Chromosomal acetylation significantly facilitates the formation of the BRD4-E2 complex, a key mediator of transcriptional repression. A specific isoform of BRD4, known as BRD4S, which lacks the CTM, interacts with the E2 protein to restrict late HPV transcription specifically in undifferentiated keratinocytes. This underscores the complex regulatory roles of BRD4 in controlling HPV transcription and its implications for HPV-associated pathologies [70].

Recent studies have deepened our understanding of how BRD4 influences the HPV infectious cycle. BRD4 is integral to the replication and transcription processes of HPV. The E2 protein of HPV binds to BRD4, tethering the viral genome to host mitotic chromosomes, which facilitates viral genome maintenance and segregation during cell division [71]. This interaction also impacts the localization of viral replication centers and influences the viral life cycle in stratified epithelia, where the virus establishes infection in basal cells and restricts the production of viral particles to differentiated cells [72].

Moreover, studies have demonstrated that BRD4 and E2 together bind to extensive regions of mitotic chromatin, enhancing the stability of the viral genome within the host cell’s nucleus throughout the lifecycle of the virus [73]. These interactions highlight the critical role of BRD4 not only in supporting HPV replication but also in its potential as a target for therapeutic strategies against HPV-related diseases.

Overall, the involvement of BRD4 in HPV biology highlights its importance in regulating viral transcription and replication, presenting it as a significant target for therapeutic interventions against HPV infections.

#### 5.2.2. HSV

Herpes simplex virus (HSV), a double-stranded DNA virus, manipulates host neuron chromatin to alternate between latency and lytic replication [74]. During lytic phases, BRD4, a BET family protein, modulates chromatin to enable the transcription of viral genes (IE, E, L) [75]. BRD4 plays a critical role in modulating HSV-1 replication through its involvement in the phosphorylation of RNA polymerase II via CDK9, enhancing the transcription of viral genes. Notably, the BET inhibitor JQ1 boosts HSV replication, indicating BRD4’s role in recruiting P-TEFb to viral promoters [76]. However, JQ1 may also disrupt the 7SK snRNP complex, releasing P-TEFb independently of BRD4 and highlighting a complex dual regulatory mechanism [77]. This suggests that both direct and indirect actions of BRD4 and related epigenetic modulators are crucial in controlling HSV transcription and could be targeted therapeutically.

#### 5.2.3. HBV

Hepatitis B virus (HBV) is a DNA virus known for its ability to cause chronic infections in the liver, leading to severe liver disease, including cirrhosis and liver cancer [78]. The virus persists in the liver in the form of covalently closed circular DNA (cccDNA), which serves as a template for viral replication and transcription [79].

BRD4 plays a significant role in the life cycle of HBV by interacting with cccDNA. BRD4 recruits the super elongation complex (SEC) to cccDNA, enhancing transcription of the viral genome [80]. Additionally, the use of BRD4 inhibitors like MS436 has shown promising results in reducing HBV transcription and destabilizing cccDNA. This highlights the potential of targeting epigenetic regulators such as BRD4 as a therapeutic strategy against HBV, particularly in overcoming drug resistance [81].

#### 5.2.4. HAdV

Human adenoviruses (HAdVs) are utilized in cancer therapy for their specificity in targeting cancer cells, particularly in treating PDAC, a notably aggressive cancer [82]. These viruses, however, face challenges such as rapid hepatic clearance and restricted replication within the tumor microenvironment [83]. Research has demonstrated that BRD4 inhibitors significantly enhance the therapeutic efficacy of HAdVs by boosting viral gene expression and replication in PDAC models [84]. Furthermore, ARGLU1, a protein involved in RNA splicing and transcription, has been shown to affect transcription and DNA damage responses. ARGLU1 influences adenovirus replication and cancer cell chemoresistance by modulating promoter-proximal RNA polymerase II pausing and interacting with key proteins like JMJD6 and BRD4 [85].

In the intricate landscape of viral infection mechanisms, BRD4 emerges as a pivotal player with multifaceted roles. The dual functionality of BRD4, acting both as a promoter of viral replication and as a therapeutic target, highlights its potential in shaping the course of viral infections.

## 6. BRD4 Proteins as Targets for Anticancer Treatment

Cancer is characterized by the profound dysregulation of gene expression, a process in which BRD4 plays a crucial role [8]. As a key member of the BET protein family, BRD4 regulates transcription by binding to acetylated histones, thereby modulating the expression of genes essential for various cellular functions and contributing to oncogenic processes. Notably, BRD4 is instrumental in the transcriptional activation of multiple oncogenes, including the well-characterized proto-oncogene Myc [33,34]. Its regulation of Myc is particularly significant as it occurs at both transcriptional and post-transcriptional levels, affecting Myc’s stability and activity within cancer cells (Table 2).

### 6.1. AML

Acute myeloid leukemia (AML) is an aggressive hematologic malignancy that significantly impacts the hematopoietic system. BRD4 plays a crucial role in the pathology of leukemia by interacting with acetylated histone tails, enhancing transcriptional processes through the recruitment of the positive transcription elongation factor b (P-TEFb) to enhancer regions [43,44]. This recruitment is essential for the phosphorylation of RNA polymerase II’s C-terminal domain, a critical step in elongating nascent mRNA. Recent studies have demonstrated that the inhibition or deletion of BRD4 can effectively hinder leukemia progression, underscoring its vital role in the disease’s pathophysiology [86]. BET proteins, including BRD4, are also known to drive leukemia by recruiting transcription factors to the promoters of oncogenes such as MYC and BCL2, establishing BRD4 as a pivotal player in leukemia’s molecular landscape [87,88].

Research has shown that JQ1, a BRD4 inhibitor, effectively targets BRD4 by suppressing the expression of MYC and other oncogenes, leading to cell cycle arrest and apoptosis in AML cells [89,90]. This suggests that BRD4 inhibition can serve as a potent therapeutic approach in managing AML by directly affecting the transcriptional misregulation that is characteristic of the disease.

### 6.2. NSCLC

Non-small cell lung carcinoma (NSCLC) is the most common lung cancer [91]. In non-small cell lung cancer (NSCLC), BRD4 exhibits upregulation, correlating with poor prognosis and aggressive cancer phenotypes such as lymph node metastasis, higher TNM stages, and poor differentiation [92]. The therapeutic inhibition of BRD4 using JQ1 and other inhibitors has shown promise in NSCLC, influencing pathways like PD-L1 expression which is crucial for immune evasion. Inhibiting BRD4 disrupts the BRD4-IRF1 complex at the PD-L1 promoter, reducing PD-L1 levels on tumor cells, and thereby enhancing the efficacy of chemoradiotherapy and PD-1 blockade [93].

BRD4 is a crucial transcriptional activator of KEAP1 in lung cancer. Specifically, BRD4 binds to the KEAP1 promoter, enhancing its transcription, which destabilizes Nrf2 [94]. This regulation suppresses Nrf2’s downstream target, glucose-6-phosphate dehydrogenase (G6PD), thereby influencing the pentose phosphate pathway (PPP) crucial for redox balance and cancer cell proliferation in small cell lung cancer (SCLC).

These findings highlight the central role of BRD4 in regulating key oncogenic pathways in NSCLC, making it a valuable target for therapeutic intervention. By integrating these mechanisms, our understanding of the complex interplay within NSCLC cells deepens, suggesting that targeting BRD4 could modulate immune responses and metabolic pathways to curb tumor growth and improve patient outcomes.

### 6.3. TNBC

Triple-negative breast cancer (TNBC) is a heterogeneous and clinically aggressive disease for which there is no targeted therapy [95]. BRD4 plays a pivotal role in the progression of triple-negative breast cancer (TNBC) by modulating various signaling pathways that drive oncogenic activities, particularly cell migration, invasion, and proliferation. Recent studies have identified the short isoform of BRD4 (BRD4-S) as notably oncogenic in TNBC, influencing extracellular-matrix-associated networks and promoting tumor dissemination through regulation of the Jagged1/Notch1 signaling pathway [96]. BRD4 binding to the proximal region of the Jagged1 promoter enhances Jagged1 expression, thereby activating Notch1 signaling, which is crucial for the epithelial–mesenchymal transition (EMT) and cancer stem cell self-renewal [97]. The selective knockdown of BRD4 suppresses Notch1 activity, thereby inhibiting TNBC cell migration and invasion. This regulatory effect of BRD4 on Jagged1/Notch1 expression underscores its potential as a therapeutic target in managing the aggressive spread of TNBC.

Furthermore, BRD4’s interaction with the AMP-activated protein kinase (AMPK) pathway introduces an additional layer of regulatory complexity in TNBC. BRD4 has been shown to downregulate AMPK, a critical enzyme in cellular energy homeostasis, which influences autophagy and apoptosis [98]. By inhibiting BRD4, there is an induced activation of AMPK, leading to autophagy-mediated cell death. This suggests that BRD4 inhibitors could also exert anticancer effects by modulating autophagy and apoptosis pathways in TNBC, offering a dual mechanism—via direct transcriptional regulation and through metabolic reprogramming—to counteract TNBC proliferation and survival [99].

In summary, the multifaceted roles of BRD4 in regulating transcriptional and metabolic pathways in TNBC present a compelling case for its targeting in therapeutic strategies.

### 6.4. NMC

NUT midline carcinoma (NMC) is a rare and aggressive form of squamous cell carcinoma that commonly arises in midline structures of the body, not specifically limited to the testis [100]. It is characterized by genetic translocations involving the NUT gene (NUTM1), most frequently forming fusions with the BRD4 or BRD3 genes [101,102]. These translocations result in the formation of a potent BRD-NUT oncoprotein. The oncoprotein disrupts normal cell differentiation and extensively modifies chromatin through increased histone acetylation, leading to the aberrant expression of c-Myc and other oncogenic drivers that promote the growth and survival of cancer cells [103].

Recent studies have shown that the BRD4-NUT fusion recruits the histone acetyltransferase p300, leading to the hyperacetylation of chromatin and activation of transcription of key oncogenes such as MYC, SOX2, and TP63. These transcription factors are associated with stem cell-like properties and contribute significantly to the aggressive nature of NMC [104]. 

Targeting the BRD4-NUT oncoprotein with BET inhibitors, such as JQ1, has demonstrated efficacy in inducing differentiation and apoptosis in NMC cells [105]. This therapeutic approach leverages the dependency of NMC cells on the oncogenic activities mediated by the BRD4-NUT fusion. BET inhibitors disrupt the interaction between the fusion protein and chromatin, effectively downregulating MYC and other critical targets, and promoting cell differentiation. 

These findings emphasize the importance of targeting the epigenetic modifications induced by the BRD4-NUT fusion and validate the use of BET inhibitors as a potential therapeutic strategy in managing NMC.

### 6.5. GBM

Glioblastoma multiforme (GBM) is the most common and aggressive type of malignant primary brain tumor [106].BRD4 as an epigenetic reader plays a critical role in tumorigenesis, including in glioblastoma (GBM) [107]. BRD4 modulates the transcription of genes that drive growth and survival in various malignancies. In GBM, BET proteins, particularly BRD4, are valuable therapeutic targets. Recent studies have documented the efficacy of BRD4 inhibitors and degraders in suppressing GBM progression in both preclinical and clinical settings [108].

BRD4 has been found to support the transcriptional activation of critical oncogenic pathways, notably by sustaining the expression of MYC and other growth-promoting genes. Moreover, recent insights have highlighted that BRD4 modulation affects the expression of immune checkpoints like PD-1 on T cells within the GBM tumor microenvironment [89]. The inhibition of BRD4 has been shown to enhance the efficacy of immunotherapies, including chimeric antigen receptor T-cell (CAR-T) therapy, by reducing the expression of immunosuppressive genes activated by BRD4 [109]. This dual role of BRD4 in both promoting oncogenic signaling and modulating immune responses presents it as a multifaceted target for GBM therapy.

## 7. BET Small-Molecule Compounds

BRD4, a key bromodomain-containing protein, plays a central role in mediating gene expression critical for the development and progression of various diseases, notably cancer. The advent of small-molecule inhibitors targeting BRD4 has opened new avenues in therapeutic development, offering potential treatments across a spectrum of diseases by interfering with crucial protein interactions that sustain disease states (Table 3).

### 7.1. Classification of BET Inhibitors

1. Pan-BET inhibitors: these inhibitors target all BET family proteins indiscriminately, affecting multiple pathways but often accompanied by side-effects such as myelosuppression and gastrointestinal toxicity due to their broad activity.
2. Selective BD1 and BD2 inhibitors: Designed to increase specificity and reduce adverse effects, these inhibitors target specific bromodomains within the BET proteins. BD1 inhibitors are typically utilized in cancer therapies, while BD2 inhibitors are more effective in modulating immune and inflammatory responses.
3. BRD4-selective inhibitors: these compounds specifically target BRD4 among BET proteins but do not differentiate between its bromodomains, potentially impacting a wide array of BRD4-mediated processes.
4. Dual-targeting inhibitors: these innovative inhibitors target BRD4 and another protein simultaneously, aiming to enhance therapeutic effects and overcome resistance common with single-agent therapies.
5. BET-PROTACs: a novel class, these bivalent molecules not only inhibit BRD4 but also promote its degradation, offering a dual mechanism that could enhance the efficacy of the therapeutic intervention.

### 7.2. Pan-BET Inhibitors

JQ1, a prominent pan-BET inhibitor, has garnered significant attention for its multifaceted therapeutic potential, particularly in oncology and virology. This small-molecule inhibitor targets the bromodomains (BD I and BD II) of BET proteins, crucial in regulating gene expression by recognizing acetylated lysines on histones. The binding of JQ1 to these bromodomains disrupts the interaction between BET proteins and chromatin, leading to altered transcriptional activity. JQ1 effectively inhibits the growth of various cancer cells by blocking the transcriptional programs essential for cancer progression, such as those regulated by the oncogene MYC [110,111]. JQ1 has shown efficacy against several viruses by impeding the BET protein-mediated transcriptional elongation of viral genes. This action results in a decreased replication and expression of viral genes, affecting viruses like HIV, HSV, HBV, HPV, SARS-CoV-2, and EBV [67,112,113,114]. I-BET151 like JQ1, an inhibitor, also targets multiple BET proteins but with slight variations in binding affinity and specificity that might influence its therapeutic application [115]. OTX015, another pan-BET inhibitor used in clinical trials, shows promising results in various hematological malignancies and solid tumors [116].

### 7.3. BD1-Selective Inhibitors

ZL0580, as a BD1-selective inhibitor, represents an innovative approach in the targeted inhibition of BET proteins, specifically focusing on the BD1 domain [35,117]. This inhibitor is notable for its role in HIV therapy and research into other viral infections. ZL0580 effectively targets the BET family of proteins, disrupting HIV transcription by specifically interfering with the interaction between CDK9 and Tat, which are critical components for HIV transcriptional elongation. This specific inhibition helps in reducing HIV viral replication. The inhibitor also promotes the formation of repressive chromatin structures at the HIV Long Terminal Repeat (LTR), contributing to the silencing of HIV transcription, thereby further suppressing the viral activity [35]. Recent studies have expanded the potential application of ZL0580, showing its effectiveness against African swine fever virus (ASFV) in porcine alveolar macrophage cells. This broad antiviral activity is characterized by dose-dependent reductions in viral titers, RNA transcription, and protein synthesis [118]. ZL0513, another BD1-selective compound, is similar in targeting specificity to ZL0580 [119]. GSK778 is part of a newer class of BET inhibitors focusing on BD1, potentially offering different biological impacts or improved safety profiles [120]. LT052 also targets BD1, and it could provide insights into the differential effects on gene expression or chromatin modulation compared to broader BET inhibitors [121].

### 7.4. BD2-Selective Inhibitors

ABBV-744 represents a significant advancement in the targeted therapy of BET bromodomains, specifically focusing on the BD2 domain, which is distinct from broader-spectrum BET inhibitors [122]. This selective approach offers enhanced therapeutic efficacy with reduced off-target effects, particularly in cancer types like acute myeloid leukemia (AML) and prostate cancer that express the androgen receptor (AR) [122,123]. Compared to broader BET inhibitors like ABBV-075, ABBV-744 exhibits fewer side-effects such as platelet and gastrointestinal toxicities, making it a safer option for long-term treatment. GSK046 is another BD2-selective inhibitor showing promise in various therapeutic settings due to its similar selective inhibition profile [124]. RVX208 specifically targets BD2 and has been studied primarily for cardiovascular diseases due to its effects on cholesterol regulation but also shows potential in cancer therapy [39].

### 7.5. Dual-Targeting Inhibitors 

NEO2734 is a dual inhibitor targeting both BET and CBP/p300 proteins, which play significant roles in oncogenic processes due to their involvement in chromatin modulation and gene transcription [125]. This inhibitor stands out because it not only targets the epigenetic regulation by BET proteins but also inhibits the histone acetyltransferase (HAT) activity of CBP and p300, which are crucial for the acetylation of histone H3 lysine 27 (H3K27ac). This dual action helps in downregulating oncogene transcription and reducing tumor progression while enhancing the immune response and overcoming drug resistance [55]. In addition, ABBV-075 is also a dual-targeting inhibitor [126].

### 7.6. BET-PROTAC

ARV-771 represents a significant advancement in the field of BET protein targeting, utilizing proteolysis-targeting chimera (PROTAC) technology to achieve the more effective degradation of BET proteins compared to traditional inhibition [127]. PROTACs like ARV-771 work by recruiting an E3 ubiquitin ligase to BET proteins, leading to their ubiquitination and subsequent degradation by the proteasome [128]. ARV-771 has shown a dramatically improved efficacy in cellular models of castration-resistant prostate cancer (CRPC) compared to traditional BET inhibitors. This is reportedly the first demonstration of a small-molecule BET degrader showing efficacy in a solid tumor malignancy, marking a potential breakthrough in cancer treatment strategies [127]. dBET1, as a PROTAC, induces the degradation of BET proteins, showing potential in overcoming resistance mechanisms that limit the efficacy of traditional inhibitors [129]. dBET6 is similar to dBET1 and also targets BET proteins for degradation but may have different pharmacokinetic properties or efficacy profiles in various cancer models [130].

The therapeutic potential of BRD4 inhibitors extends beyond cancer treatment. These inhibitors have been explored for their efficacy in inflammation, cardiovascular diseases, and as part of antiviral strategies. Ongoing research continues to refine these inhibitors to enhance their specificity and reduce side-effects. The development of next-generation BET inhibitors, particularly those that can selectively target different aspects of BRD4’s activity, holds promise for more effective and tailored therapies. This includes the development of compounds like PROTACs that offer a mechanism for not just inhibiting BRD4 function but also reducing its cellular levels, potentially leading to more robust and lasting therapeutic effects.

## 8. Conclusions

The BET family of proteins, particularly BRD4, has emerged as a pivotal regulator in both the innate immune response to viral infections and gene transcription modulation under these conditions. The utilization of small-molecule inhibitors targeting these proteins has demonstrated substantial potential, extending from virology to oncology. However, despite these promising developments, the intricate mechanisms by which BET proteins influence these processes remain only partially understood. This knowledge gap underscores the critical need for enhanced research efforts aimed at unraveling the complex regulatory roles of the BET family.

Future investigations should focus on the detailed dissection of the molecular interactions and regulatory networks modulated by BET proteins in diverse pathological settings. It is imperative to delineate how these proteins interact with other cellular machinery and influence gene expression at a granular level. Such an understanding is crucial not only for refining current therapeutic approaches but also for pioneering novel strategies that could potentially target a wide array of viral pathogens and cancer types.

In conclusion, the study of the multifaceted roles of the BET family, especially BRD4, represents a dynamic and promising field of research. Continued advancements in this area are expected to catalyze significant breakthroughs in medical science, potentially revolutionizing therapeutic options and profoundly impacting public health. By addressing the underlying mechanisms of BET protein function and their implications in disease, we can pave the way for innovative treatments that leverage these critical regulatory pathways.

## Figures and Tables

**Table 1:** BET family protein interactions with viruses. BET family proteins are closely associated with different types of infections and affect viral infections through different mechanisms of action.

Virus	BET	BET Domain	Viral Protein/Gene	Functions
RSV	BRD4	BDs	-	Modulates NF-kB and IRF transcription factors within the RLR pathway, enhancing transcription of immune response genes, particularly type I and III interferons and cytokines crucial for antiviral defense.
HIV	BRD4	CTD, BDs	Tat, LTR	BRD4 promotes HIV-1 latency by competitively inhibiting P-TEFb-mediated transcription induced by the virus-encoded Tat protein, playing a key role in the regulation of viral transcription and latency.
SARS-CoV-2	BRD2; BRD4	BDs, ET	Spike (S) and Envelope (E) proteins	BRD4 interacts with the acetylated C-terminus of the SARS-CoV-2 E protein, which could facilitate viral entry into host cells.
HPV	BRD4	CTD, PDID, BID	E2	BRD4 interacts with the E2 protein of HPV, influencing transcriptional regulation of the viral genome and affecting the lifecycle, including viral replication and cell cycle progression.
HSV	BRD4	BDs	P-TEFb, RNAP II	BRD4 modulates the phosphorylation of RNA Pol II via CDK9, enhancing viral gene expression and impacting the immune response during viral infection.
HBV	BRD4	-	cccDNA	Enhances transcription from cccDNA by recruiting the super elongation complex (SEC), influencing viral transcription and replication.
HAdV	BRD4	-	-	BRD4’s role in adenovirus infections includes influencing transcriptional regulation of the viral genome.

**Table 2:** The role of BET family proteins in the development of different cancers.

Cancer	BET Inhibitor	BET Interaction	BRD4 Role
AML	JQ1; OTX015; ACC010	acetylated histone tail; transcription factors and chromatin modifiers; MYC, BL2 promoter	BRD4 modulates the expression of crucial oncogenes like MYC, enhancing cell proliferation and survival in AML.
NSCLC	JQ1; IBET-151; MZ1	NF-κB target genes	BRD4 influences cell cycle regulation and survival by modulating the expression of genes such as MYC and PD-L1, which are critical for tumor progression and immune evasion.
TNBC	JQ1; SDU-071;	transcriptional regulators and chromatin remodeling complexes; PD-L1;NF-κB	BRD4 supports oncogenic programs in TNBC by enhancing the transcription of genes driving cell proliferation and metastasis.
NMC	OTX015; NEO2734	acetylated histones	NMC is characterized by the BRD4-NUT fusion, where BRD4’s transcriptional regulatory activities are hijacked to drive aggressive tumor growth.
GBM	JQ1; IBET151; OTX015; MZ1	enhancer regions and its role in chromatin remodeling	BRD4’s interaction with enhancer regions and its role in chromatin remodeling are critical for maintaining the aggressive phenotypes of GBM.

**Table 3:** The role of different types of BET family inhibitors in related diseases.

Type	Inhibitors	Pharmacological Action	Related Disease	Factors Involved
Pan-BET inhibitors	JQ1	Antivirus; Anti-inflammation; Antitumor	HIV; HPV; HSV;RSV; HBV; SARS-CoV-2; ASFV; cancers like NUT carcinoma, AML	NF-kB; RIG-I; cGAS-STING; c-Myc; RelA; P-TEFb
	IBET-151	Antivirus; Anti-inflammation; Antitumor	PRV; HSV; Glioma; Melanoma; Neuroblastoma,; Multiple myeloma; Osteoarthritis; Rheumatoid arthritis etc.	NF-kB; Myc; JUN; RELA; IRF3
	OTX015	Antivirus; Antitumor;	HIV; PRV; HSV; SARS-CoV-2; AML; hematological malignancies; advanced solid tumors; glioblastoma multiforme etc.	NF-kB; Myc; IRF3; RELA; P-TEFb
BD1 selective inhibitors	ZL0580	Antitumor; Antivirus	HIV; ASFV	P-TEFb
	ZL0513	Antivirus; Anti-inflamation	RSV	RELA; IRF1; IRF7; IRF3
	GSK778	Antitumor	AML	-
	LT052	Antitumor	Prostate cancer	AR
BD2 selective inhibitors	GSK046	Anti-inflammation	-	MCP-1
	ABBV-744	Antivirus; Antitumor	SARS-CoV-2; AML; Myelofibrosis	-
	RVX208	Cardioprotection	Pulmonary arterial hypertension(PAH); Diabetes mellitus(DM)	FOXM1
Dual-targeting inhibitors	NEO2734	Antitumor	Solid tumors; drug-resistant cancers	SECs
	ABBV-075	Antitumor	Prostate cancer; hematological malignancies and solid tumors	-
BET-PROTAC	ARV-771	Antitumor	CRPC (castration-resistant prostate cancer)	-
	dBET1	Anti-inflammation; Antitumor	AML; inflammation	c-Myc; E3; NF-κB
	dBET6	Anti-inflammation; Antitumor	Neuroinflammation; glioma	cGAS-STING; E2F

## Data Availability

No new data were created or analyzed in this study.
